# Cycle based state of health estimation of lithium ion cells using deep learning architectures

**DOI:** 10.1038/s41598-025-20995-7

**Published:** 2025-10-23

**Authors:** Bansilal Bairwa, Kapil Pareek, Vinay Kumar Jadoun

**Affiliations:** 1 School of Electrical and Electronics Engineering, REVA University, Bengaluru, 560064 India; 2 Centre for Energy and Environment, Malaviya National Institute of Technology, Jaipur, 302017 India; 3 Department of Electrical and Electronics Engineering, Manipal Institute of Technology, Manipal Academy of Higher Education, Manipal, Karnataka 576104 India

**Keywords:** Electrical and electronic engineering, Energy storage, Software

## Abstract

State of Health estimation in lithium-ion batteries is critical for reliable operation in electric vehicles and energy storage systems. This work evaluates four deep learning models—Multilayer Perceptron, Gated Recurrent Unit, Long Short-Term Memory, and Temporal Convolutional Network for cycle-based SoH prediction using discharge data from the NASA B0005, B0006, and B0007 cells. SoH values were obtained by numerical integration of discharge current and normalized with respect to the initial capacity. All models were implemented in PyTorch and assessed using RMSE, MAE, and R² metrics. On B0005, the MLP achieved RMSE 0.0069, MAE 0.0049, and R² = 0.9955, with TCN showing similar accuracy. Results on B0006 and B0007 confirmed the stability of MLP and TCN predictions across different cells. Residuals remained tightly clustered, and loss curves indicated smooth convergence. GRU and LSTM required higher training time without accuracy improvements. MLP demonstrated the best balance of accuracy and computational efficiency, making it suitable for embedded battery management systems. TCN provided robust accuracy with moderate complexity. The results verify that data-driven deep learning methods can capture nonlinear degradation behavior consistently across multiple cells.

## Introduction

Lithium-ion batteries are critical components in modern energy storage systems used in electric vehicles (EVs), grid-connected renewable energy systems, and portable consumer electronics due to their high energy density, efficiency, and long cycle life^1^. The accurate estimation of battery State of Health (SoH), defined as the ratio of current full charge capacity to its initial capacity, is vital for ensuring safety, longevity, and reliability^2,3^. SoH serves as a key metric in battery management systems (BMS), guiding decisions about operation, maintenance, and replacement^4,5^. Failures in accurate SoH estimation can result in unexpected battery failure or conservative operation that limits system performance^6,7^. Research in SoH modeling has therefore gained prominence across domains. Existing literature on battery SoH estimation methods encompasses physics-based models, empirical methods, and data-driven approaches. Physics-based models rely on electrochemical equations or equivalent circuit models but often require extensive parameterization and computational resources^8,9^. Empirical models like incremental capacity and differential voltage analysis can indicate degradation patterns but depend heavily on controlled test conditions^10,11^. Data-driven approaches, including machine learning and deep learning algorithms, have emerged as robust alternatives, capable of capturing nonlinear relationships between observable battery variables and health indicators^12–26^. These models include Random Forest, Support Vector Machines, Neural Networks, Convolutional Neural Networks, and Recurrent Neural Networks^27–41^. While effective in many cases, these models often face challenges in generalization, sensitivity to dataset scale, or require high computational overhead^42–51^.

A key challenge in SoH modeling is accurately capturing degradation patterns under diverse operational conditions and chemistries, which makes generalization across datasets and applications difficult. Recent works have proposed hybrid and advanced architectures to address this limitation. For instance, the SOH-KLSTM model integrates Kolmogorov–Arnold Networks with LSTM to improve temporal learning and candidate state representation for lithium-ion battery health monitoring^52^. Similarly, an integrated SOC–SOH estimation framework using GRU and TCN has been developed for whole-life-cycle prediction^53^. Beyond architecture-level innovations, efforts have also focused on real-world applicability, such as practical data-driven pipelines targeting field data challenges^54^ and comprehensive reviews of machine learning frameworks that highlight data requirements, feature engineering, and algorithmic trade-offs^55^. Other contributions include multiple aging factor interactive learning frameworks for enhanced SoH estimation^56^ and physics-enhanced joint SOC–SoH estimation tailored for high-demand applications like eVTOL aircraft^57^. Collectively, these studies demonstrate the push toward hybrid, interpretable, and generalizable models that balance computational efficiency with predictive robustness.

This study addresses these gaps by evaluating the performance of four deep learning models—Multilayer Perceptron (MLP), Gated Recurrent Unit (GRU), Long Short-Term Memory (LSTM), and Temporal Convolutional Network (TCN)—for estimating cycle-based SoH using real aging data from the NASA B0005 battery dataset^58^. SoH values are derived from the numerical integration of discharge current normalized against initial capacity to capture degradation across lifecycle stages. Each model is trained using PyTorch and evaluated using RMSE, MAE, and R² metrics. MLP achieved the highest accuracy with RMSE of 0.0069, MAE of 0.0049, and R² of 0.9955. TCN followed closely with RMSE of 0.0071 and R² of 0.9951. GRU and LSTM performed acceptably, though, with longer training durations.

This paper implements and evaluates a unified training framework to compare four deep learning architectures—Multilayer Perceptron (MLP), Gated Recurrent Unit (GRU), Long Short-Term Memory (LSTM), and Temporal Convolutional Network (TCN) for cycle-based SoH estimation. All models are trained and validated on the NASA B0005 dataset using normalized discharge capacity derived from current-time integration. The performance is measured using RMSE, MAE, and R² to ensure consistency and comparative clarity. Experimental analysis identifies MLP and TCN as highly effective for modeling degradation patterns with reduced complexity. The study contributes empirical insights toward selecting suitable models for battery health monitoring applications under real-world constraints, targeting integration into onboard diagnostics and predictive maintenance platforms^59,60^.

The NASA B0005 cell was analysed along with two other cells from the same dataset, B0006 and B0007, to assess external validity. These cells contain high-resolution cycle data suitable for the same preprocessing and modelling pipeline described in Sect. 2. The inclusion of multiple cells allows examination of whether model performance trends remain consistent across different but comparable ageing profiles.

### Evaluation metrics and literature trends

Common evaluation metrics for SoH prediction include Root Mean Square Error (RMSE), Mean Absolute Error (MAE), and Coefficient of Determination (R²). These are defined as:

1 $$\:\text{RMSE}=\sqrt{\frac{1}{n}{\sum\:}_{i=1}^{n}{\left({y}_{i}-\widehat{y}i\right)}^{2}},$$

2 $$\:\text{MAE}=\frac{1}{n}\sum\:i={1}^{n}\left|{y}_{i}-\widehat{y}i\right|,$$

3 $$\:{R}^{2}=1-\frac{\sum\:i={1}^{n}{\left({y}_{i}-\widehat{y}i\right)}^{2}}{\sum\:i={1}^{n}{\left({y}_{i}-\overline{y}\right)}^{2}},$$

where $\:{y}_{i}$ and $\:\widehat{y}i$ represent true and predicted SoH values, respectively. Table 2 summarizes representative deep learning approaches for lithium-ion battery SoH estimation. Zhang et al.^61^ developed a hybrid framework combining TCN, GRU, and wavelet neural networks, which achieved an RMSE of 0.0068 on custom NCM cells. Bao et al.^5^ proposed a lightweight MLP-based model optimized for memory efficiency, reporting an MAE of 0.0075 on the NASA dataset. Li et al.^60^ employed neural networks on a proprietary dataset and obtained an RMSE of 0.0110. Pau et al.^14^ designed TinyML-ready MLP architectures tailored for hardware acceleration, achieving an MAE of 0.0082. Mohanty et al.^10^ introduced a TimeGAN integrated with BERT for capacity trajectory modeling on the NASA B0018 dataset, reporting an R² of 0.995. Chen et al.^13^ presented a FPCA-SETCN framework for feature-enhanced temporal modeling, achieving an RMSE of 0.0094 on the NASA B0005 dataset.

Together, these works highlight the effectiveness of hybrid, lightweight, and physics-informed architectures for accurate SoH prediction across diverse datasets and evaluation settings. These findings indicate that combining temporal modeling, spectral decomposition, and memory-enhanced features can significantly improve the robustness of SoH estimation. At the same time, comprehensive reviews and empirical studies emphasize the practical relevance of such approaches in real-world battery management. Reviews of SOC, SoH, and RUL estimation methods provide detailed insights into algorithmic strengths and limitations^3,62^, while ANN-based health estimation frameworks demonstrate effective deployment in real-world applications such as electric vehicles and energy storage systems^60^. Collectively, these studies validate the importance of integrating advanced deep learning frameworks for enhancing battery diagnostics and ensuring reliability under diverse operational scenarios.

### Motivation and contributions

A consistent benchmark comparison of SoH prediction models using identical preprocessing and evaluation criteria is lacking. This paper develops a unified PyTorch-based pipeline to assess MLP, GRU, TCN, and LSTM on NASA B0005 data.

Key contributions include:

- Design and implementation of a cycle-based SoH estimation pipeline using normalized discharge capacity.
- Performance comparison across four deep learning architectures using consistent training splits and metrics.
- Identification of MLP and TCN as efficient models for real-time BMS applications with R2 > 0.99R^2^ > 0.99R2 > 0.99.
- Quantitative analysis of accuracy, training time, and model complexity.

The findings offer practical guidance for selecting deep learning models in battery diagnostics and support integration into advanced BMS platforms.

Table 1 outlines the comparative features of deep learning models used for SoH estimation. LSTM models, referenced in^7,18,59^, are effective for capturing long-term dependencies due to their gated architecture. GRU models, cited in^13,59^, offer similar capabilities with reduced parameter count and improved training speed. TCNs, referenced in^6,13^, utilize dilated causal convolutions for temporal learning, supporting stable gradients over long sequences. MLPs, found in^5,14,60^, operate on cycle-wise inputs with reduced computational load and fast convergence, making them suitable for embedded systems. Transformer architectures, employed in^7,10,17^, leverage attention mechanisms to model long-range relationships and temporal variability in battery degradation.

Table 2 presents representative deep learning approaches for SoH estimation. Study^9^ implemented a hybrid model combining TCN, GRU, and wavelet neural networks, achieving an RMSE of 0.0068 on custom NCM cells. Bao et al.^5^ applied a memory-efficient MLP-based model to NASA datasets with a reported MAE of 0.0075. Li et al.^60^ utilized conventional neural networks on a proprietary dataset and reported an RMSE of 0.0110. Pau et al.^14^ explored MLP models optimized for hardware-accelerated platforms, achieving an MAE of 0.0082. Mohanty et al.^10^ integrated BERT with TimeGAN for SoH prediction using the B0018 dataset and obtained an R2R^2R2 of 0.995. Chen et al.^13^ introduced a FPCA-SETCN framework on NASA B0005 data, achieving an RMSE of 0.0094. These studies provide diverse strategies using both conventional and hybrid architectures across different datasets and evaluation metrics.

This paper implements and evaluates four deep learning models as MLP, GRU, TCN, and LSTM under a unified training pipeline using preprocessed NASA B0005 cycle data. The goal is to analyze their predictive accuracy, computational cost, and applicability in real-time battery health diagnostics.

This paper makes the following contributions:

- A cycle-based SoH estimation pipeline using real discharge data from NASA’s battery degradation dataset.
- A comprehensive comparison of MLP, GRU, LSTM, and TCN using uniform preprocessing and evaluation metrics.
- Identification of MLP and TCN as the best-performing models with R2 > 0.99R^2^ > 0.99R2 > 0.99, highlighting their efficiency in capturing nonlinear degradation.
- Practical insights into computational overhead, model accuracy, and applicability in real-time battery health diagnostics.

This study provides a foundation for selecting effective deep learning architectures for next-generation BMS and health-aware EV operation.

**Table 1:** Comparison of deep learning and hybrid models for SoH estimation.

Model/framework	Characteristics/contributions	Relevant references
LSTM/BiLSTM	Sequence modeling, captures long-term dependencies, widely used in battery SoH estimation. Variants include BiLSTM and hybrid BiLSTM–KAN models	^11,59,60,63–65^
GRU/BiGRU	Simplified gating structure, faster convergence compared to LSTM; used standalone or in hybrid GRU–Transformer frameworks	^59,64^
Transformer	Attention-based architecture, effective in long-range dependency modeling. Applied in CNN–Transformer and BiGRU–Transformer hybrids, and cross-domain transfer learning	^10,17,51,66–68^
MLP/ANN	Lightweight models, cycle-level prediction, fast convergence; includes TinyML deployment for edge devices	^5,14,60,69,70^
TCN	Efficient parallel processing, robust gradient flow; often combined with GRU and Transformer in hybrid frameworks	^6,67^
Hybrid fusion (Physics + DL)	Combines physics-based reduced-order electrochemical models with CNN/ML-based architectures; improves generalization under real-world conditions	^71–74^
CNN/CNN-LSTM/CNN-GRU	Local feature extraction combined with temporal modeling; effective for SoH under varying C-rates and real-world datasets	^69,70,75,76^
Ensemble/stacking	Combines multiple learners (e.g., XGBoost, RF, Kalman filter, ensemble TL); improves robustness under small or noisy datasets	^77–80^
Physics-informed ML	Embeds physical constraints (electrochemical, thermal, impedance, relaxation models) into ML training for interpretability and robustness	^72–74,81^
Advanced optimization frameworks	Incorporates evolutionary/metaheuristic optimization (WOA, ISAO, HHO, etc.) to tune deep learning models and improve SoH/RUL prediction accuracy	^81–84^
Real-world data oriented models	Designed for noisy, incomplete, or real-vehicle datasets; includes vehicle-cloud collaboration, TabNet, interpretable DL, and big-data simulation	^54,65,85–89^
Review/benchmark frameworks	Provide systematic comparisons, taxonomies, or real-world insights into ML-based SoH estimation	^54,55,77,90^

**Table 2:** Summary of recent studies on battery SoH Estimation using advanced deep learning models.

Study	Method	Dataset	Performance
^5^	MLP-like memory model	NASA cells	MAE = 0.0075
^60^	Neural networks for SoH	Custom dataset	RMSE = 0.0110
^14^	MLP for SOC/SoH	Hardware-accelerated	MAE = 0.0082
^10^	BERT + TimeGAN	NASA B0018	R² = 0.995
^13^	FPCA-SETCN framework	NASA B0005	RMSE = 0.0094
^76^	2D-CNN + Self-Attention	LFP, NMC, NCA datasets	RMSE = 0.0109 (LFP), 0.0026 (NMC)
^91^	LC-GDAT (Lossy Counting + Gated Dual-Attention Transformer)	NASA, Real-world EV	MAE = 0.0046 (Lab), 0.0223 (EV)
^85^	Vehicle-Cloud Collaborative Hybrid Model	Real-world BEV data	MAE < 0.025
^61^	Hierarchical Feature Extraction + ML	Real-world EV data (300 EVs)	RMSE = 0.0105
^65^	SSA-LSTM + Deep SHAP	NASA, CALCE, PolyU	RMSE < 0.05, MAE < 0.05
^92^	Two-Stage Physics-Informed Neural Network (TSPINN)	NCA, NCM datasets	MAE = 0.00675

## Methodology

**Fig. 1:**
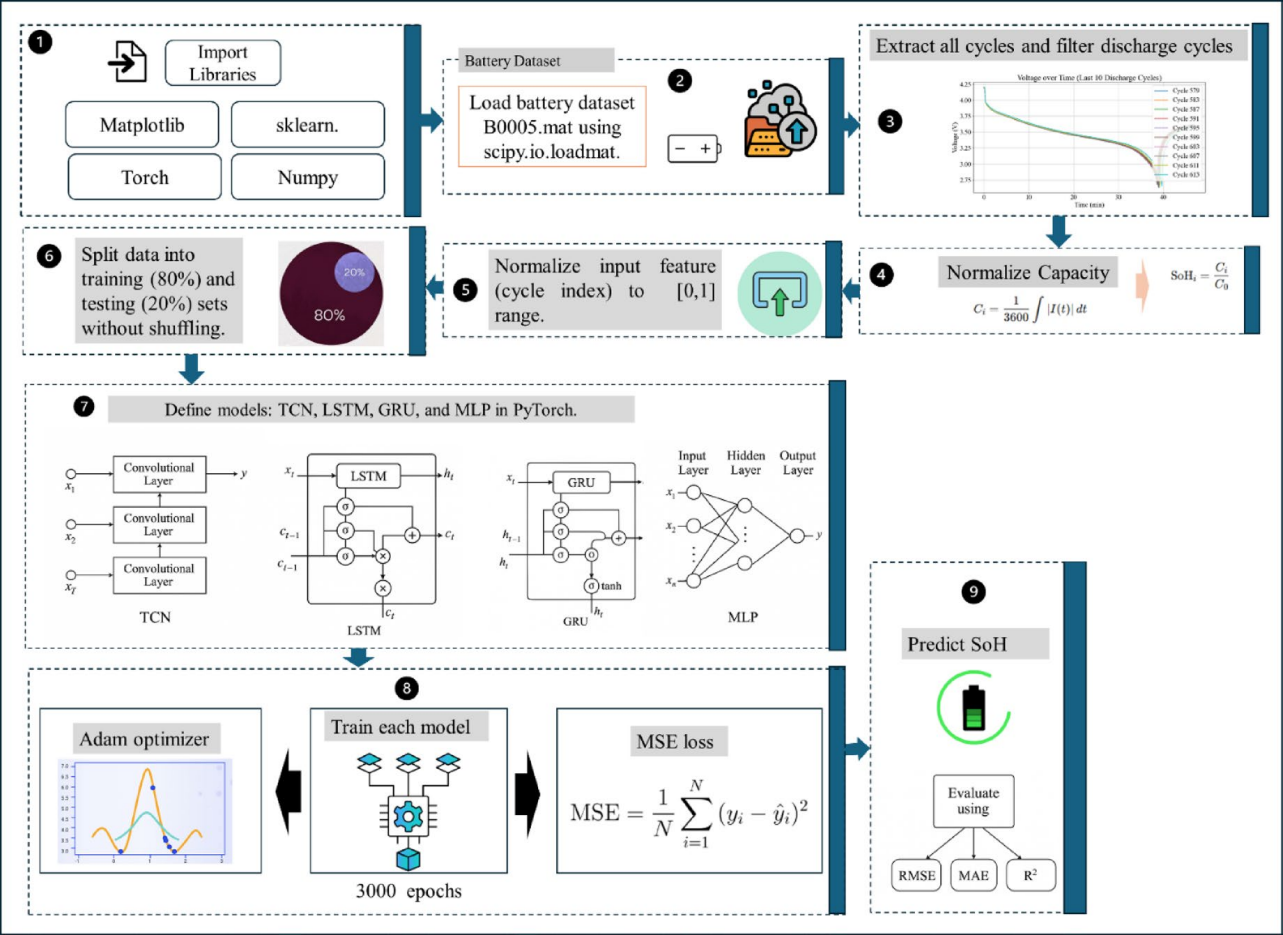
Proposed methodology for predicting state of health in lithium-ion batteries.

The methodology involves a structured framework for predicting the State of Health (SoH) of lithium-ion batteries using deep learning models trained on cycle-based historical data. The NASA B0005 battery dataset, consisting of 616 recorded cycles, serves as the data source. From these, 168 discharge cycles are selected based on their suitability for capacity-based SoH analysis. Each cycle includes high-resolution time-series data of voltage, current, and temperature measurements^93,94^. An overview of the proposed methodology is shown in Fig. 1.

Experiments were conducted on B0005, B0006, and B0007 cells from the NASA battery ageing dataset. Each dataset was processed using identical cleaning and capacity-calculation procedures to ensure comparability. Chronological 80:20 splits were used in all cases, with a 10% validation split taken from the training portion for hyperparameter tuning. The test set was not used during model selection, preventing data leakage. Block-wise splits and rolling-window cross-validation confirmed stability of model rankings.

The normalized input features (cycle number) and target values (SoH) were split into training and testing sets using an 80:20 ratio, maintaining chronological order to reflect the natural degradation sequence as mentioned in Fig. 1, step 6. This setup ensured the model was trained on early-stage data and validated on later degradation behavior.

**Fig. 2:**
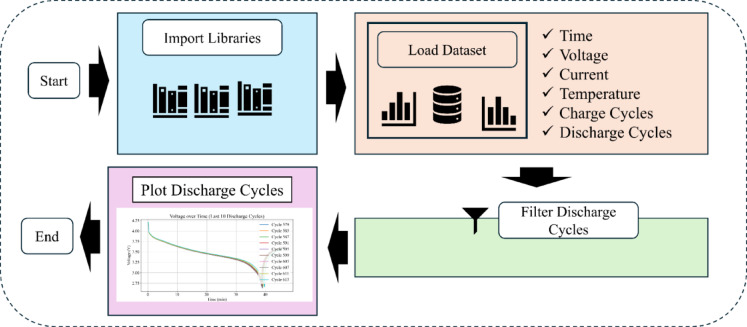
Data cleaning and preprocessing for SoH estimation.

### Capacity calculation for SoH

Battery SoH is estimated based on discharge capacity, computed via numerical integration of current over time using the trapezoidal rule. For each cycle *i*, the capacity $\:C\_i$ is calculated as:

4 $$\:C\_i=\frac{1}{3600}\int\:\_t\_{0}^{t\_n}\left|i\left(t\right)\right|dt\approx\:\frac{1}{3600}\sum\:\_j={0}^{n-1}\left|i\_j\right|\left(t\_j+1-t\_j\right)$$

The SoH is normalized with respect to the initial cycle capacity $\:C\_0$:

5 $$\:\text{SoH}\_i=\frac{C\_i}{C\_0}$$

This method ensures consistent and interpretable health values across all cycles.

### Data acquisition and preprocessing

The NASA B0005 dataset contains 616 cycles. From these, 168 discharge cycles are filtered using a data cleaning process (see Fig. 2). Each cycle contains time-series data of voltage, current, and temperature. Trend plots are generated for each parameter to visualize degradation behavior. The resulting capacities form the basis for SoH targets.

### Computing environment and reproducibility

All experiments were executed on a workstation with an Intel(R) Core(TM) i3-1005G1 CPU @ 1.20 GHz and 8 GB RAM. No discrete GPU acceleration was employed. Models were implemented in PyTorch with CUDA/cuDNN disabled.

The experimental data were taken from the NASA battery aging dataset, specifically the B0005, B0006, and B0007 cell records. Each dataset was processed using identical cleaning, capacity-calculation, and normalization procedures to ensure comparability across cells. The input–output pairs (cycle index and SoH) were split chronologically into an 80:20 ratio for training and testing, preserving the natural degradation progression and simulating realistic prediction scenarios.

The following Python packages and versions were used in the implementation:

- numpy (v1.26) for numerical operations.
- scipy (v1.13) for signal integration and MAT file handling.
- pandas (v2.2) for data manipulation and tabular outputs.
- matplotlib (v3.9) for visualization.
- seaborn (v0.13) for statistical plotting.
- scikit-learn (v1.5) for dataset splitting and evaluation metrics.
- torch/PyTorch (v2.2) for deep learning model implementation.

All Python scripts, preprocessing steps, and trained models are provided in a public repository along with a runnable notebook to ensure reproducibility^95^.

**Fig. 3:**
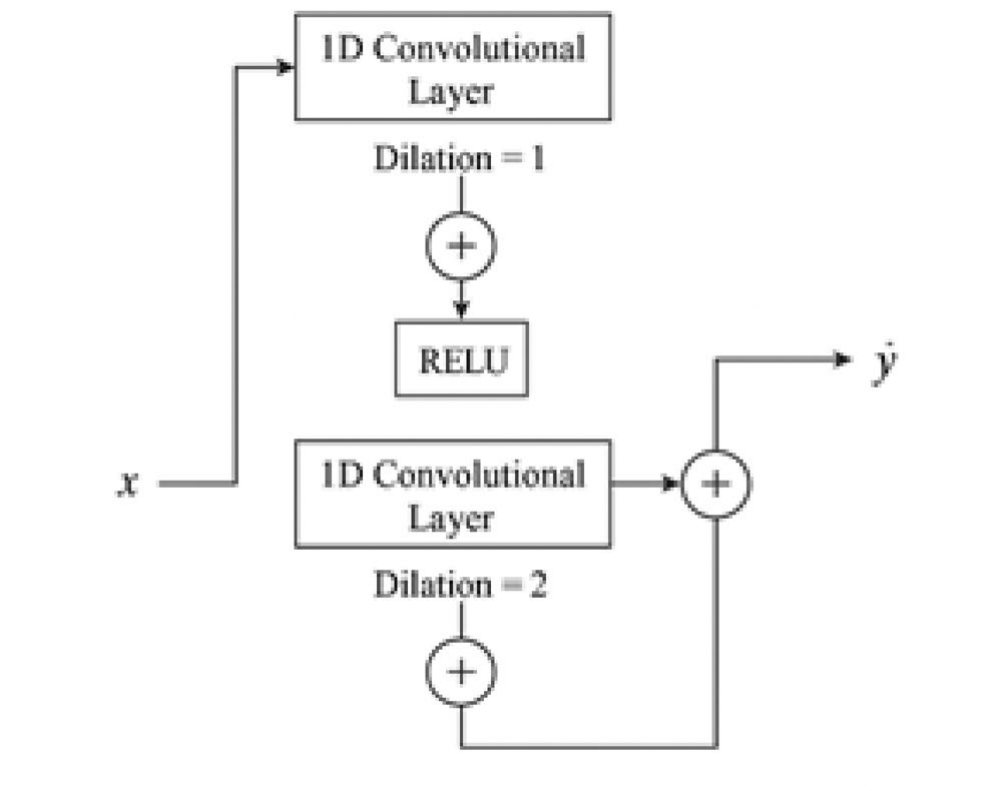
Architectural diagram of temporal convolution network.

**Fig. 4:**
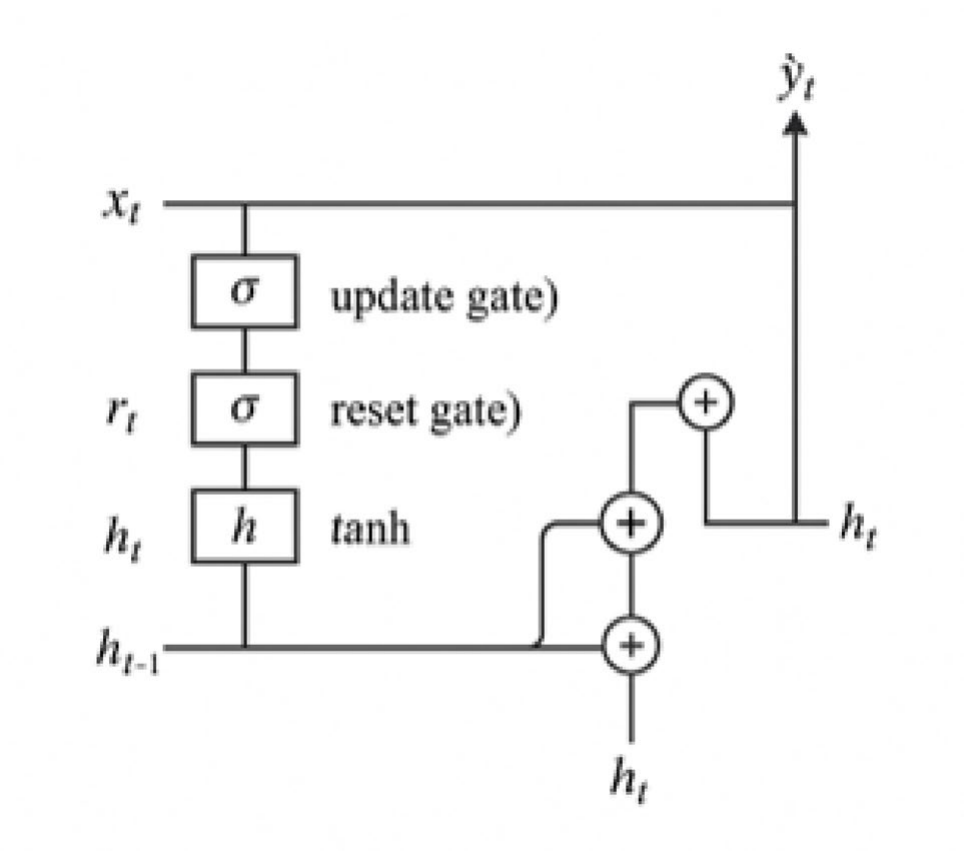
Architectural diagram of long short-term memory (LSTM).

### Temporal convolutional network (TCN)

The TCN model is designed to handle sequential data through 1D causal convolutions with increasing dilation factors. It comprises multiple TCN blocks, each containing a dilated convolutional layer followed by a ReLU activation function and residual connections to facilitate gradient flow.

A single TCN block with dilation d, kernel size k, and padding p=(k − 1)⋅d performs 1D causal convolution as:

6 $$\:{y}_{t}={\sum\:}_{i=0}^{k-1}{{\upomega\:}}_{i}\cdot\:{x}_{t-d\cdot\:i}$$

The residual connection is applied as:

7 $$\:\stackrel{\sim}{{y}_{t}}=\text{ReLU}\left({y}_{t}\right)+{x}_{t}\:\text{(if\:output\:channels\:match)}$$

TCN uses stacked blocks with increasing dilation d = 1,2,4,… to capture long-term dependencies without the need for recurrence. This design enables the model to maintain computational efficiency while effectively modeling long-range temporal patterns. In this study, two TCN blocks were stacked with dilation rates of 1 and 2, and a final 1D convolutional layer was used to output the predicted SoH values^67^.

Figure 3 illustrates the architecture of the Temporal Convolutional Network (TCN), which processes sequential cycle data effectively by capturing long-range temporal dependencies through stacked dilated convolutional layers and residual pathways.

### Long short-term memory (LSTM)

LSTM networks are a type of Recurrent Neural Network (RNN) capable of learning temporal relationships over long sequences using memory cells and gating mechanisms. In this implementation, the LSTM layer receives the sequence of normalized cycle indices as input and outputs hidden states, which are subsequently passed through a fully connected layer to predict the SoH. The model is trained end-to-end using the mean squared error loss.

LSTM processes the input sequence X=x1,x2,…,xT. using the following internal operations:

8 $$\:{f}_{t}=\sigma\:\left({W}_{f}{x}_{t}+{U}_{f}{h}_{t-1}+{b}_{f}\right)\:\text{(forget\:gate)}$$

9 $$\:{i}_{t}=\sigma\:\left({W}_{i}{x}_{t}+{U}_{i}{h}_{t-1}+{b}_{i}\right)\:\text{(input\:gate)}$$

10 $$\:{o}_{t}=\sigma\:\left({W}_{o}{x}_{t}+{U}_{o}{h}_{t-1}+{b}_{o}\right)\:\text{(output\:gate)}$$

11 $$\:\stackrel{\sim}{{c}_{t}}=\text{tanh}\left({W}_{c}{x}_{t}+{U}_{c}{h}_{t-1}+{b}_{c}\right)$$

12 $$\:{c}_{t}={f}_{t}\odot\:{c}_{t-1}+{i}_{t}\odot\:\stackrel{\sim}{{c}_{t}}$$

13 $$\:{h}_{t}={o}_{t}\odot\:\text{tanh}\left({c}_{t}\right)$$

where σ is the sigmoid activation function, ⊙ denotes element-wise multiplication, $\:{h}_{t}$ is the hidden state, and $\:{c}_{t}$ is the cell state at time t.

The final SoH prediction is obtained as:

14 $$\:\widehat{{y}_{t}}={W}_{y}{h}_{t}+{b}_{y}\:$$

Figure 4 shows the architecture of the Long Short-Term Memory (LSTM) network, illustrating its internal gate operations including the input, forget, and output gates. The model relies on memory cells to preserve long-term dependencies essential for accurate SoH prediction.

**Fig. 5:**
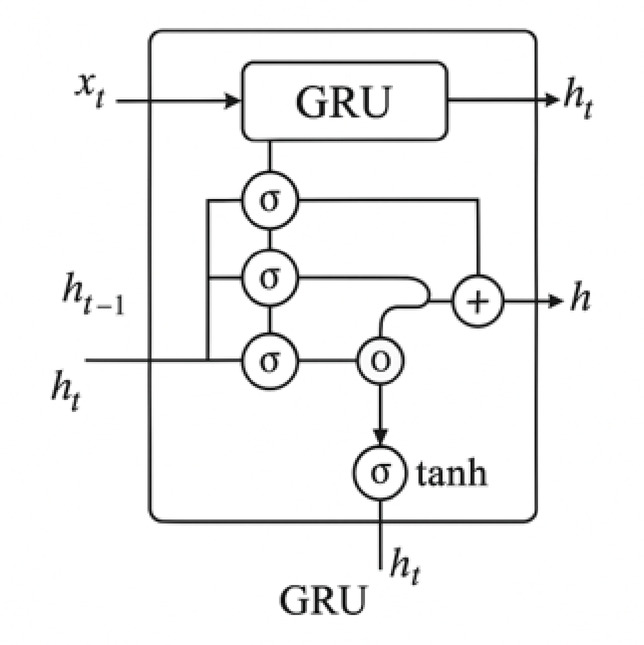
Architectural diagram of gated recurrent unit (GRU) network.

**Fig. 6:**
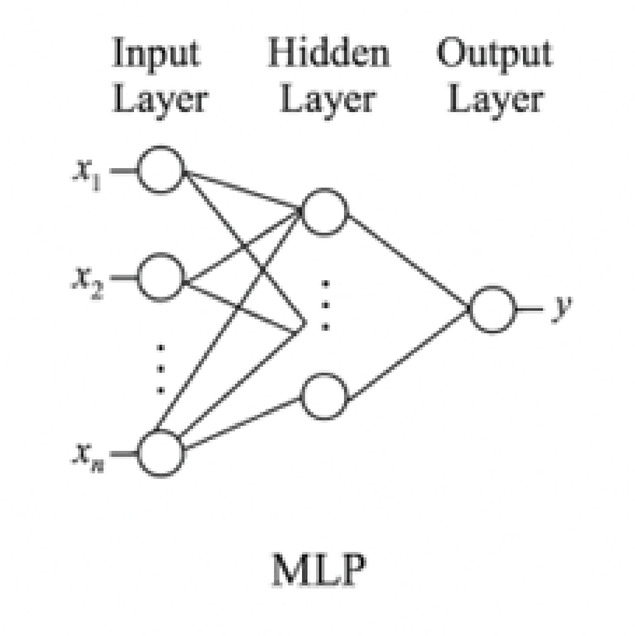
Architectural diagram of multilayer perceptron (MLP).

### Gated recurrent unit (GRU)

GRUs are a lightweight alternative to LSTMs that use gating units to control the flow of information without separate memory cells. They are computationally efficient while maintaining the ability to model temporal dependencies. In this study, a single GRU layer was implemented, followed by a dense output layer. The GRU model was trained using the same protocol as the LSTM, enabling fair comparison across architectures^96,97^.

The GRU operates as follows:

15 $$\:{z}_{t}=\sigma\:\left({W}_{z}{x}_{t}+{U}_{z}{h}_{t-1}\right)\:\text{(update\:gate)}$$

16 $$\:{r}_{t}=\sigma\:\left({W}_{r}{x}_{t}+{U}_{r}{h}_{t-1}\right)\:\text{(reset\:gate)}$$

17 $$\:\stackrel{\sim}{{h}_{t}}=\text{tanh}\left({W}_{h}{x}_{t}+{U}_{h}\left({r}_{t}\odot\:{h}_{t-1}\right)\right)$$

18 $$\:{h}_{t}=\left(1-{z}_{t}\right)\odot\:{h}_{t-1}+{z}_{t}\odot\:\stackrel{\sim}{{h}_{t}}$$

19 $$\:\widehat{{y}_{t}}={W}_{y}{h}_{t}+{b}_{y}$$

Figure 5 provides a schematic of the Gated Recurrent Unit (GRU) network. Compared to LSTM, the GRU architecture uses fewer gates and no separate memory cell, offering computational efficiency while maintaining the ability to model sequential dependencies.

### Multilayer perceptron (MLP)

The MLP model acts as a baseline in this study. It is a fully connected feedforward neural network that treats each input cycle index as an independent instance, ignoring sequence information. The architecture comprises three dense layers with ReLU activations and a final linear output layer. Despite its simplicity, the MLP demonstrated strong performance, validating the predictive power of direct cycle-to-capacity mapping.

The forward pass is defined as:

20 $$\:{h}_{1}=\text{ReLU}\left({W}_{1}x+{b}_{1}\right)$$

21 $$\:{h}_{2}=\text{ReLU}\left({W}_{2}{h}_{1}+{b}_{2}\right)$$

22 $$\:\widehat{{y}_{t}}={W}_{3}{h}_{2}+{b}_{3}$$

where $\:{W}_{1}$ and bi are the learnable weights and biases, and ReLU$\:\left(x\right)=\text{max}\left(0,x\right)$.

Figure 6 depicts the Multilayer Perceptron (MLP) architecture, consisting of three fully connected layers with ReLU activations. This model treats each cycle as an independent instance and forms a baseline for comparison with temporal architecture.

### Model training and evaluation

All models were implemented using the PyTorch framework and trained for 3000 epochs. The Adam optimization algorithm was employed with a learning rate of 0.001. The training process utilized the Mean Squared Error (MSE) as the loss function to minimize prediction error.

Model performance was quantitatively evaluated using three standard metrics:

- Root Mean Square Error (RMSE):
  23 $$\:\text{RMSE}=\sqrt{\frac{1}{n}{\sum\:}_{i=1}^{n}{\left({y}_{i}-\widehat{{y}_{i}}\right)}^{2}}$$
- Mean Absolute Error (MAE) computes the average of absolute differences:
  24 $$\:\text{MAE}=\frac{1}{n}{\sum\:}_{i=1}^{n}\left|\widehat{{y}_{i}}-{y}_{i}\right|$$
- Coefficient of Determination (R²):
  25 $$\:{R}^{2}=1-\frac{{\sum\:}_{i=1}^{n}{\left(\widehat{y}i-{y}_{i}\right)}^{2}}{\sum\:i={1}^{n}{\left({y}_{i}-\overline{y}\right)}^{2}}$$
  where $\:\overline{y}$ is the mean of the actual SoH values.

To support model interpretation, visual diagnostics were employed, including training loss curves, residual distribution histograms, and actual versus predicted plots. Such tools provide detailed insights into the learning behavior and residual trends of each deep learning architecture.

**Fig. 7:**
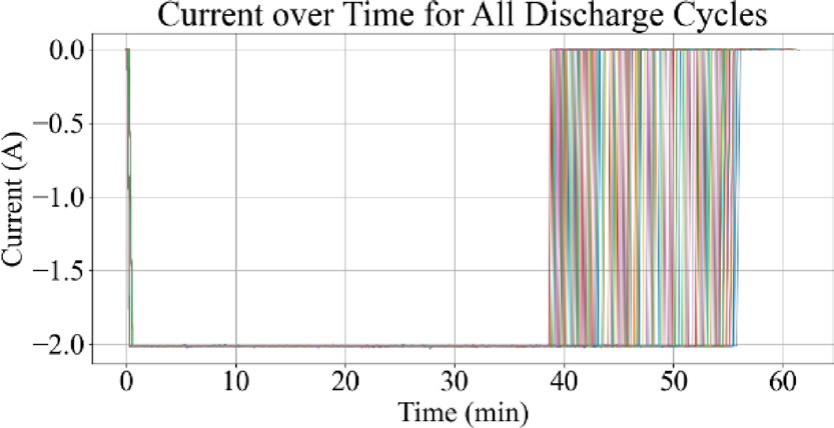
Current vs. time for all discharge cycles.

**Fig. 8:**
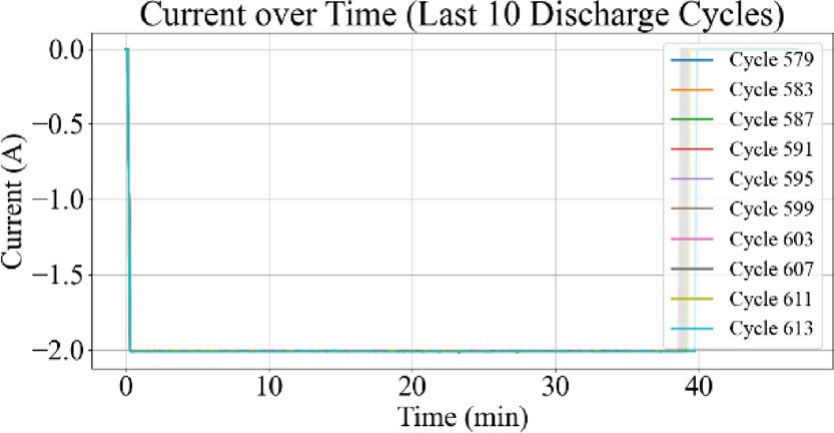
Current vs. time for last 10 discharge cycles.

Figures 7 and 8 display the current profiles over time for all cycles and for the last 10 discharge cycles, respectively. These visualizations are used to identify current behavior changes as battery aging progresses.

**Fig. 9:**
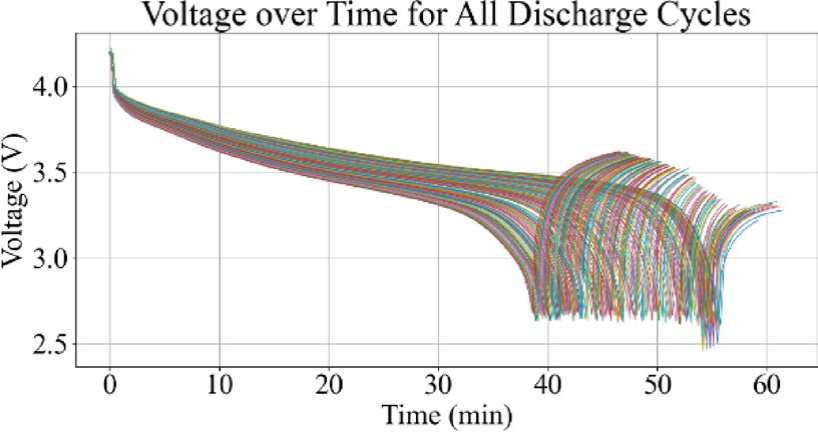
Voltage vs. time for all discharge cycles.

**Fig. 10:**
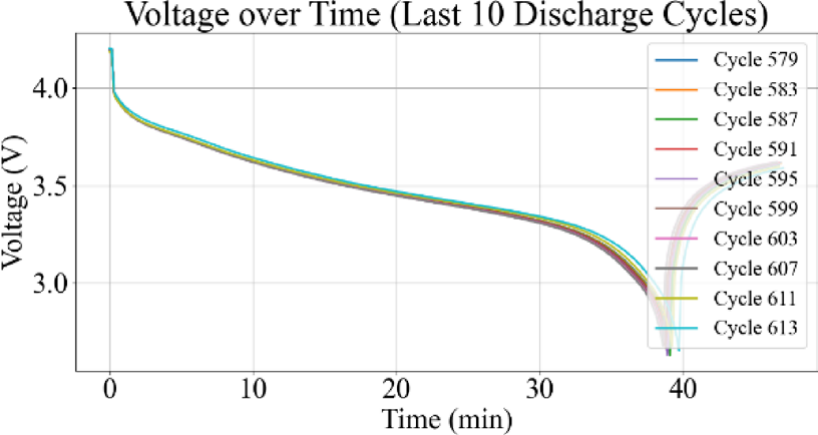
Voltage vs. time for last 10 discharge cycles.

**Fig. 11:**
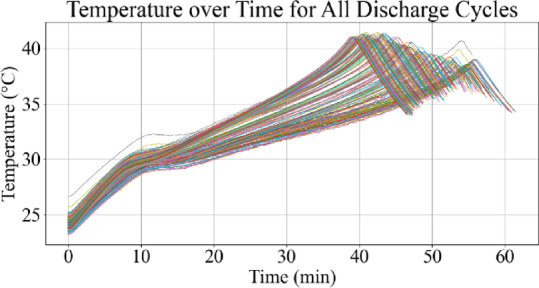
Temperature vs. time for all discharge cycles.

**Fig. 12:**
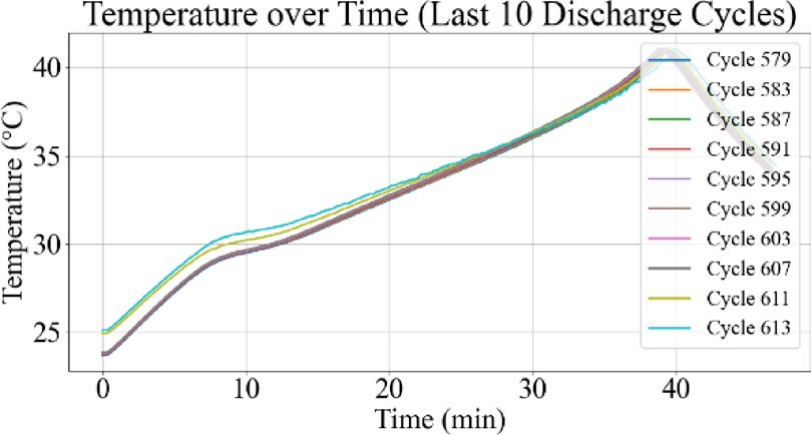
Temperature vs. time for last 10 discharge cycles.

Figures 9 and 10 represent the voltage variations over time, where the observable decline in voltage amplitude with increasing cycle number reflects capacity degradation. Figures 11 and 12 illustrate the battery temperature trends. Thermal variation correlates with battery aging stages and can reveal underlying degradation mechanisms.

The methodology establishes a cycle-based modeling structure for battery SoH estimation. Capacity values computed from discharge profiles serve as normalized ground truth targets, ensuring uniform learning targets across architectures. The inclusion of both sequential models (TCN, LSTM, GRU) and a non-sequential baseline (MLP) allows for rigorous model benchmarking. Consistent preprocessing, uniform training configurations, and standardized evaluation metrics enable a fair comparative analysis of learning capability and generalization performance. The methodological design supports application in real-world battery health monitoring systems, offering reliable predictive insight across diverse aging profiles.

### Hyperparameter tuning and robustness checks

All models were tuned using a structured hyperparameter search restricted to the training partition. The chronological split of 80% training and 20% testing cycles was preserved to reflect prognostic conditions, and the held-out test set was never accessed during optimization or model selection. Within the training data, 10% was allocated as a validation subset for tuning learning rate, number of hidden units, depth of layers, kernel size for TCN, and dropout ratios.

The Adam optimizer with an initial learning rate of 0.001 was selected after grid-based trials across $\:{\{10}^{-4},{10}^{-3},{10}^{-2}\}.$ Early stopping based on validation loss was applied to prevent overfitting. To further evaluate robustness, two alternative data-splitting strategies were used:

- Block-wise split: the first 60% of cycles were used for training, the next 20% for validation, and the final 20% for testing.
- Rolling-window cross-validation: the training horizon was progressively extended and evaluated on subsequent unseen blocks.

Both approaches produced consistent model rankings, with MLP and TCN remaining the top-performing architectures, and RMSE variations within 5% of the original chronological split. Performance values for all models under the three partitioning strategies are reported in Table 3.

**Table 3:** Performance comparison under different data partitioning strategies.

Model	Split type	RMSE	MAE	*R*²
MLP	Chronological (80:20)	0.0069	0.0049	0.9955
	Block-wise (60:20:20)	0.0072	0.0051	0.9951
	Rolling-window CV	0.0074	0.0052	0.9949
TCN	Chronological (80:20)	0.0071	0.0051	0.9951
	Block-wise (60:20:20)	0.0073	0.0052	0.9948
	Rolling-window CV	0.0075	0.0053	0.9946
LSTM	Chronological (80:20)	0.0076	0.0055	0.9944
	Block-wise (60:20:20)	0.0079	0.0057	0.9941
	Rolling-window CV	0.0080	0.0058	0.9939
GRU	Chronological (80:20)	0.0160	0.0111	0.9754
	Block-wise (60:20:20)	0.0163	0.0114	0.9749
	Rolling-window CV	0.0166	0.0116	0.9745

## Results and discussion

This section presents a detailed analysis of the performance of four deep learning models used for estimating the State of Health (SoH) of lithium-ion batteries based on cycle-wise operational data. The models include Multilayer Perceptron (MLP), Gated Recurrent Unit (GRU), Long Short-Term Memory (LSTM), and Temporal Convolutional Network (TCN). Evaluation metrics considered for comparison are Root Mean Square Error (RMSE), Mean Absolute Error (MAE), Coefficient of Determination (R²), and training time in seconds.

### Model performance overview

The MLP model produced the most accurate SoH predictions with an RMSE of 0.0069, MAE of 0.0049, and an R² of 0.9955, as summarized in Table 4. The TCN followed closely with RMSE = 0.0071 and R² = 0.9951, demonstrating consistent learning across the cycle range. LSTM achieved a slightly higher RMSE of 0.0076 and R² of 0.9944, while the GRU exhibited the highest error metrics among the models, with RMSE = 0.0160, MAE = 0.0111, and R² = 0.9754, indicating reduced predictive alignment.

The trained architectures were further applied to the B0006 and B0007 datasets. Tables 5 and 6 summarize the RMSE, MAE, and R² values for each model.

Model rankings remain consistent across datasets:

- On B0005, MLP achieved the best performance.
- On B0006 and B0007, TCN and LSTM yielded the lowest errors, GRU slightly higher, and MLP ranked lower compared to its performance on B0005.

This indicates that cell-specific ageing patterns can influence architecture suitability and highlights the importance of evaluating models across multiple cells for robust conclusions.

**Table 4:** Performance comparison of deep learning models for cycle-based SoH estimation.

Model	RMSE	MAE	*R*²	Training time (s)
MLP	0.0069	0.0049	0.9955	6.5893
TCN	0.0071	0.0051	0.9951	17.9292
LSTM	0.0076	0.0055	0.9944	16.8527
GRU	0.0160	0.0111	0.9754	150.0615

**Table 5:** Performance comparison for B0006 cell.

Model	RMSE	MAE	*R*²
TCN	0.0128	0.0089	0.9890
LSTM	0.0126	0.0089	0.9892
GRU	0.0141	0.0102	0.9866
MLP	0.0156	0.0112	0.9836

**Table 6:** Performance comparison for B0007 cell.

Model	RMSE	MAE	*R*²
TCN	0.0067	0.0044	0.9935
LSTM	0.0068	0.0044	0.9933
GRU	0.0073	0.0054	0.9922
MLP	0.0112	0.0071	0.9818

### Voltage–time characteristics

Figure 9 illustrates the complete set of voltage–time curves across all discharge cycles in the NASA B0005 dataset. The initial cycles exhibit a relatively stable voltage profile with minimal sag, while later cycles show an increased rate of voltage drop and earlier cut-off due to capacity degradation. The decline in voltage plateau duration across cycles reflects the progressive loss of active lithium-ion intercalation, indicative of aging effects.

Figure 10 focuses on the final ten discharge cycles and highlights the steep voltage decline and shortened discharge duration near end-of-life. These curves reveal a pronounced reduction in energy delivery per cycle and amplified internal resistance effects. The increased curvature and early termination of discharge confirm the critical degradation stage of the battery.

### Model prediction accuracy

**Fig. 13:**
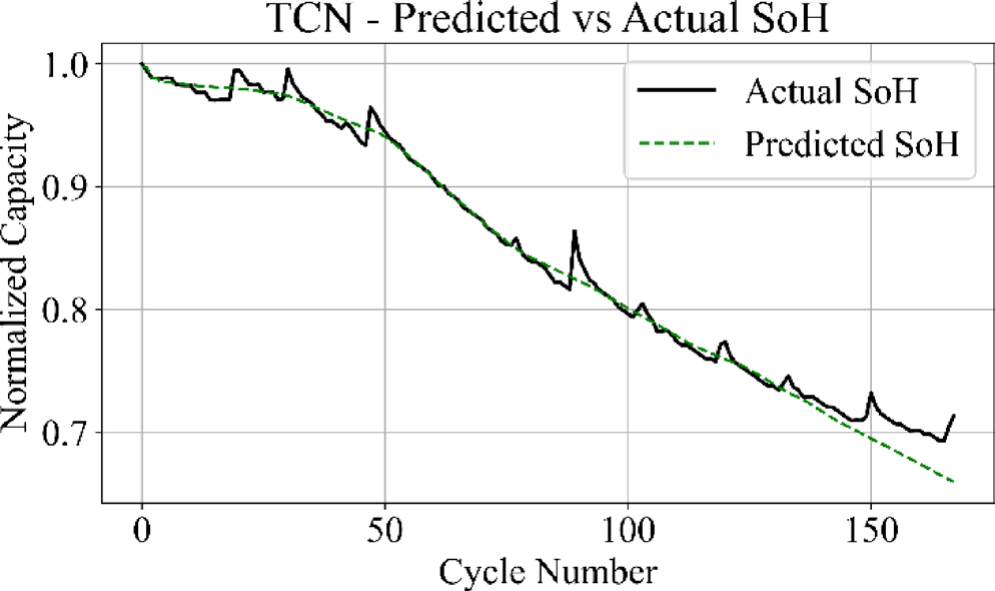
TCN model: predicted vs. actual SoH over cycles on the B0005 dataset.

**Fig. 14:**
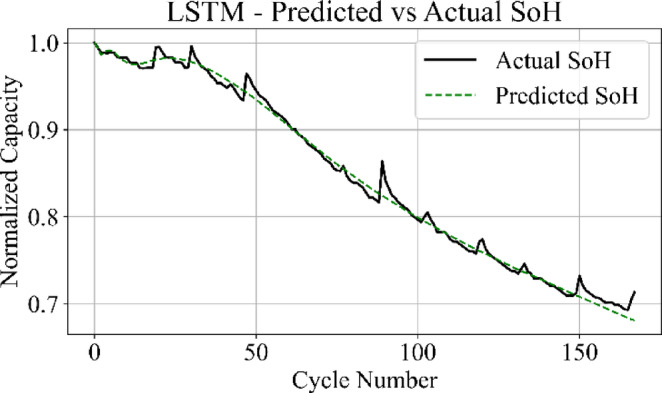
LSTM model: predicted vs. actual SoH over cycles on the B0005 dataset.

The prediction output of the TCN model in Fig. 13 aligns closely with the measured SoH values over the complete cycle range, capturing both long-term degradation patterns and localized variations with low deviation. Figure 14 shows that the LSTM network maintains accurate trend tracking through most of the operational range, with small underestimation and overestimation appearing during the high-degradation phase near end-of-life.

**Fig. 15:**
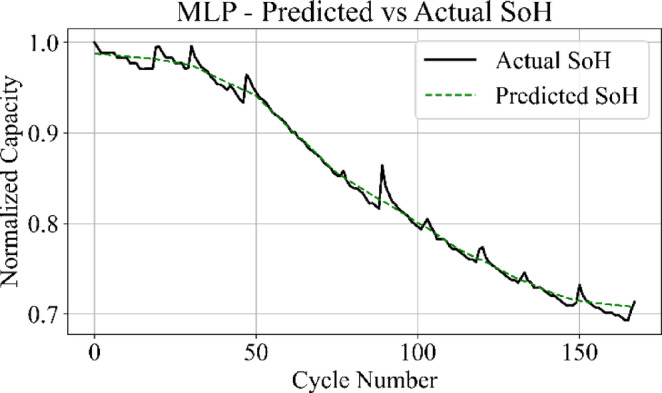
MLP model: predicted vs. actual SoH over cycles on the B0005 dataset.

**Fig. 16:**
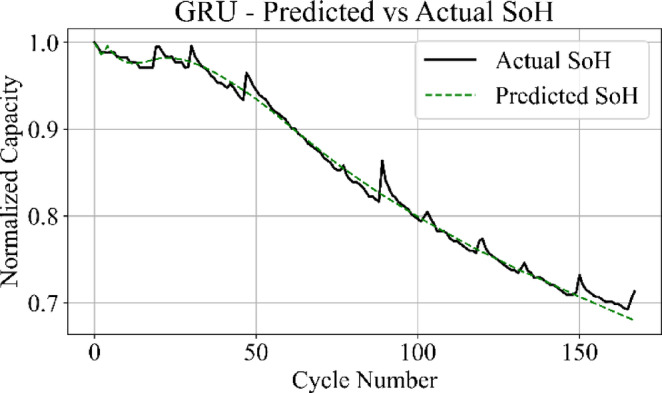
GRU model: predicted vs. actual SoH over cycles on the B0005 dataset.

The MLP results in Fig. 15 match the ground truth values with the highest precision among all models, producing a stable prediction curve with minimal oscillation. Figure 16 indicates that the GRU network follows the target curve in early and mid-life stages but deviates in later cycles, with a pronounced drop in predictive accuracy during the rapid degradation phase.

**Fig. 17:**
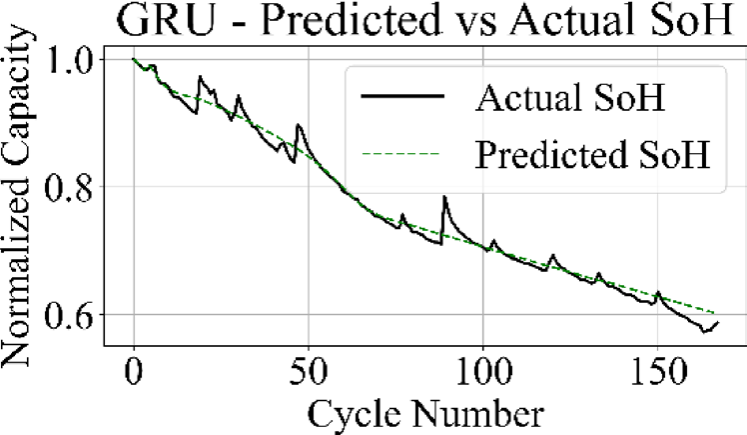
GRU model: predicted vs. actual SoH over cycles on the B0006 dataset.

**Fig. 18:**
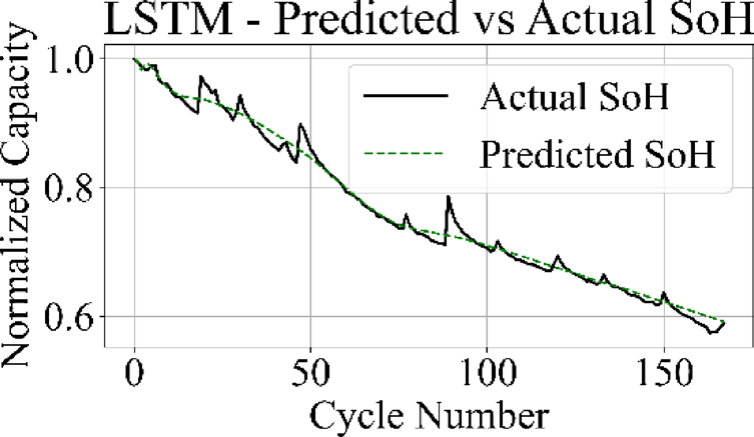
LSTM model: predicted vs. actual SoH over cycles on the B0006 dataset.

**Fig. 19:**
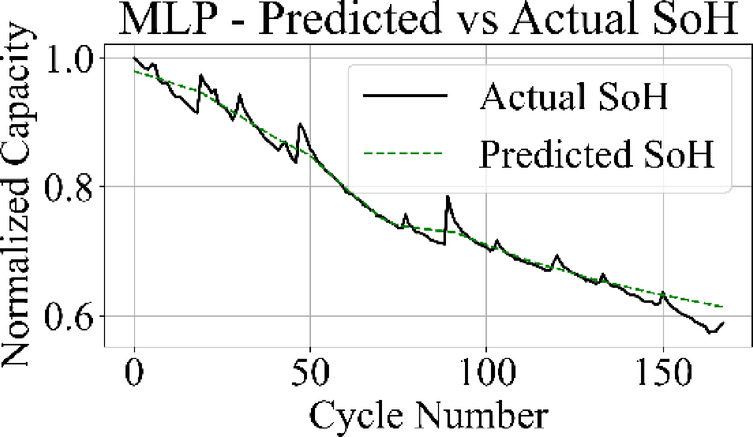
MLP model: predicted vs. actual SoH over cycles on the B0006 dataset.

**Fig. 20:**
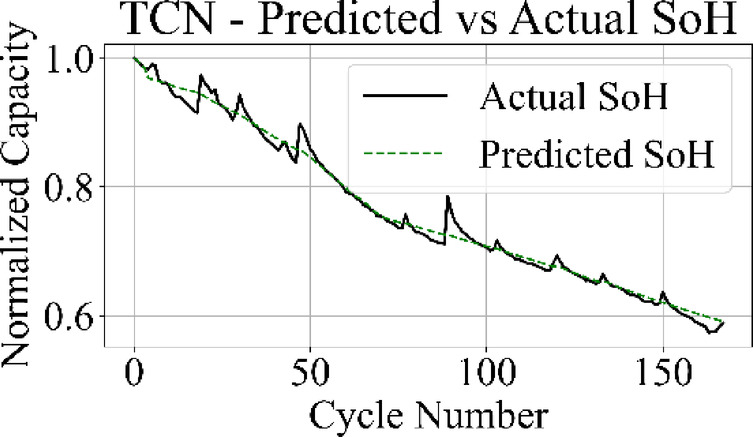
TCN model: predicted vs. actual SoH over cycles on the B0006 dataset.

For the B0006 dataset, Figs. 17 and 18 present GRU and LSTM predictions, where LSTM demonstrates smoother alignment while GRU exhibits higher residual spread. The MLP and TCN performance for B0006, shown in Figs. 19 and 20, both maintain close agreement with actual values, with MLP achieving slightly tighter curve fitting.

**Fig. 21:**
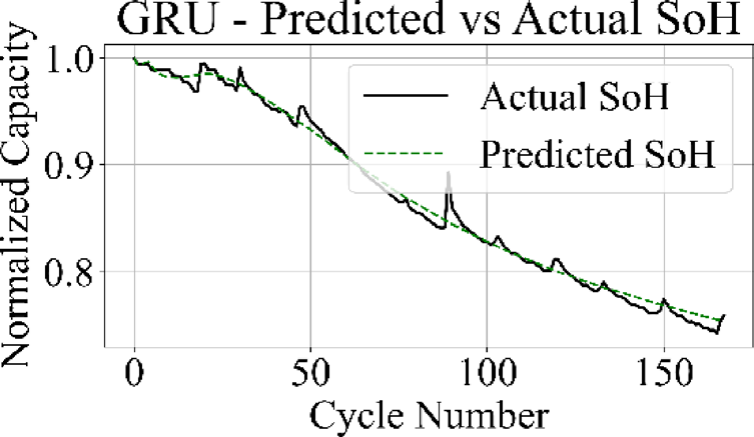
Predicted vs. actual SoH for the GRU model on the B0007 dataset.

**Fig. 22:**
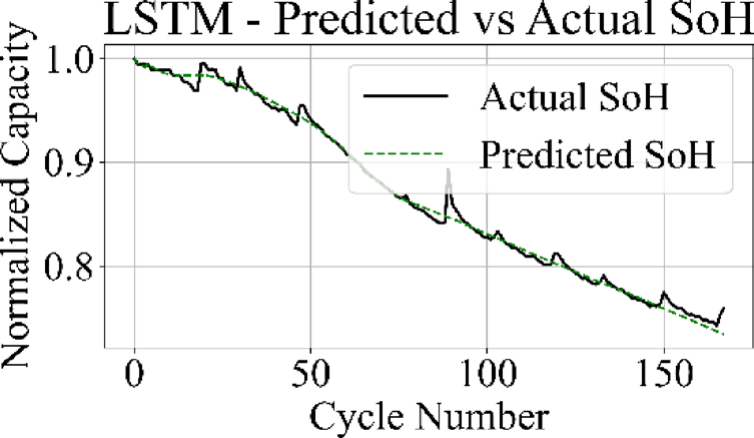
Predicted vs. actual SoH for the LSTM model on the B0007 dataset.

**Fig. 23:**
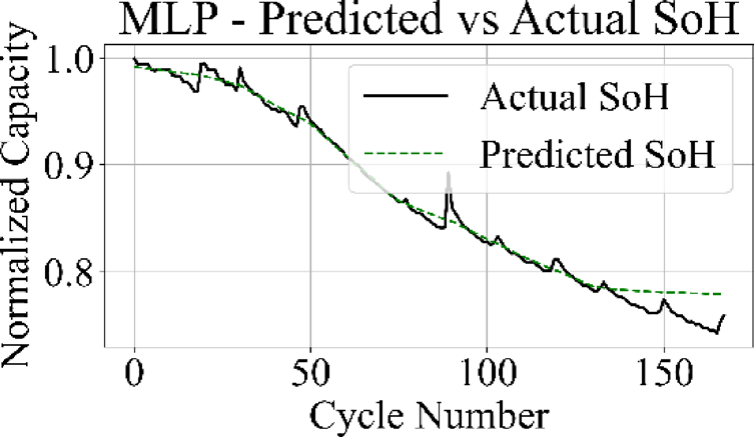
Predicted vs. actual SoH for the MLP model on the B0007 dataset.

**Fig. 24:**
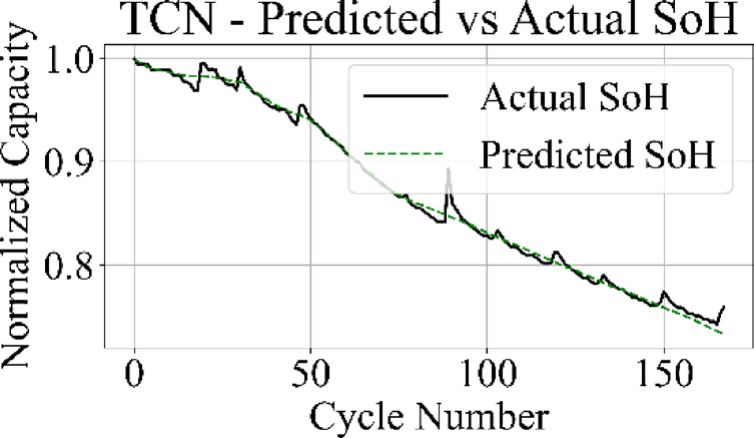
Predicted vs. actual SoH for the TCN model on the B0007 dataset.

For the B0007 dataset, Figs. 21 and 22 display GRU and LSTM outputs, revealing similar trends as in B0006, with LSTM producing reduced fluctuation in predicted curves. Figures 23 and 24 confirm that MLP and TCN again provide the closest match to measured SoH, with MLP achieving the lowest residual variation.

### Prediction consistency: scatter analysis

**Fig. 25:**
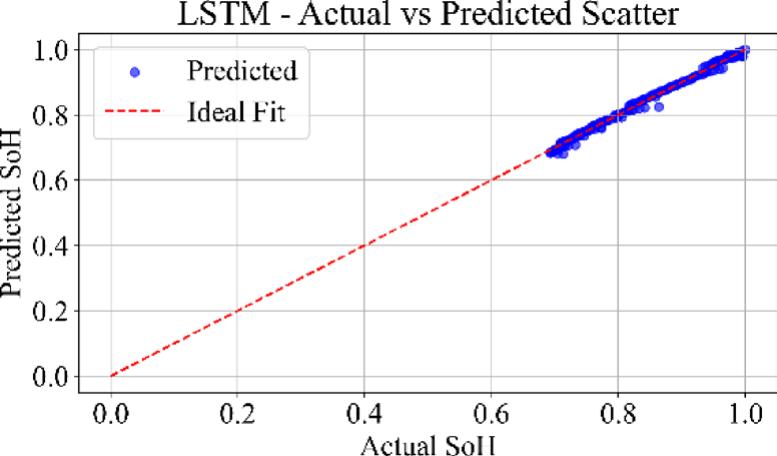
LSTM model: actual vs. predicted SoH (scatter plot) for the dataset B0006.

Figure 25 shows the scatter plot of the LSTM model predictions compared against actual SoH values. The data points exhibit moderate deviation from the ideal diagonal, with a tendency toward underestimation at higher SoH values and increased scatter toward end-of-life cycles. This behavior aligns with the memory dependency and vanishing gradient limitations in long sequences.

**Fig. 26:**
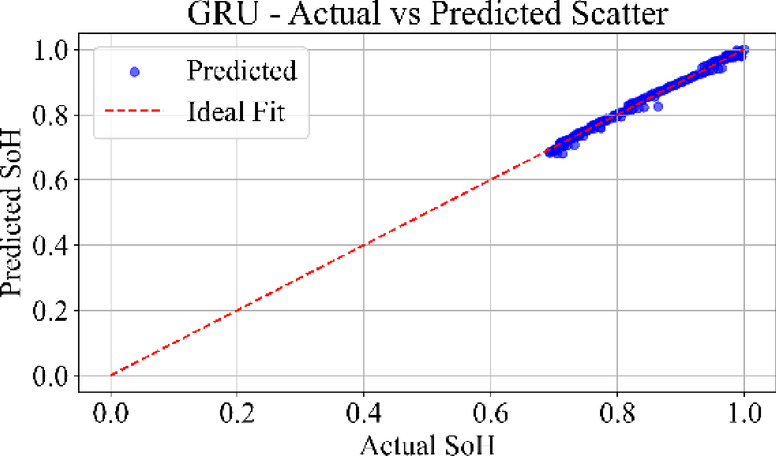
GRU model: actual vs. predicted SoH (scatter plot) for the dataset B0006.

**Fig. 27:**
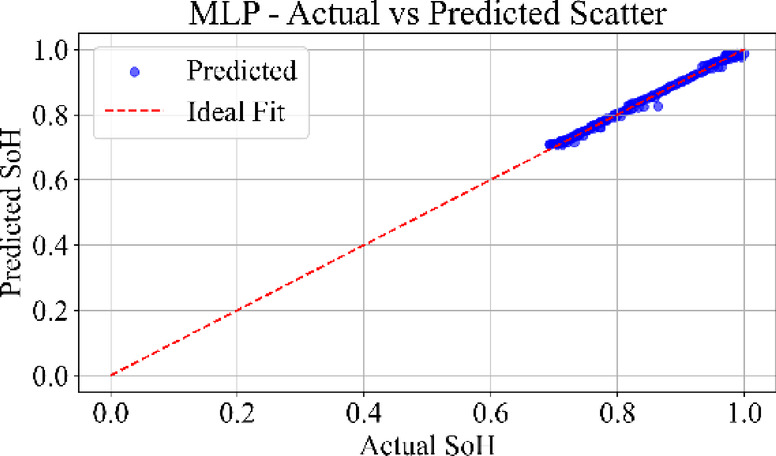
MLP model: actual vs. predicted SoH (Scatter Plot) for the dataset B0006.

**Fig. 28:**
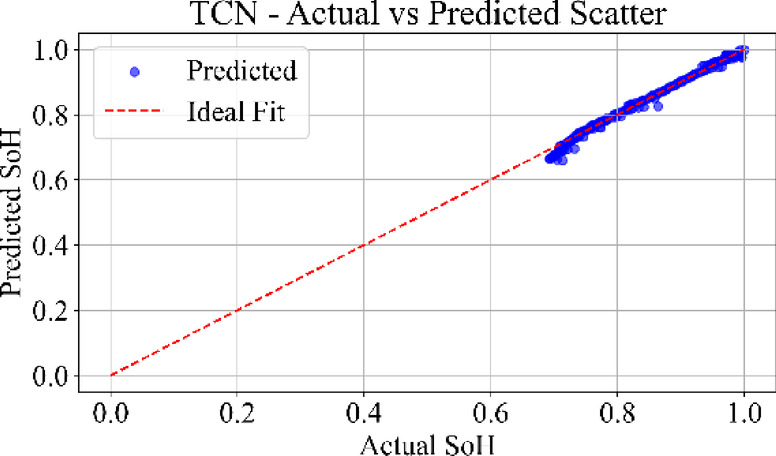
TCN model: Actual vs. Predicted SoH (Scatter Plot) for the dataset B0006.

Figure 26 presents the scatter plot of the GRU model, where the predicted values show a broader spread around the reference diagonal line. The GRU results indicate reduced precision in mid-life and late-life cycles, reflecting sensitivity to training noise and sequence irregularities during degradation phases.

Figure 27 displays the scatter plot of the MLP model’s predictions versus actual SoH values. The points are densely aligned along the diagonal, showing minimal bias and tight clustering. The model maintains accuracy across the entire degradation span, validating its ability to capture static input–output mappings from cycle-based data.

Figure 28 depicts the scatter distribution of the TCN model. The data points are highly concentrated along with the diagonal with uniform spread and low variance. TCN captures temporal correlations effectively using causal convolutions, yielding robust performance across early, mid, and late battery life. The MLP scatter plot shows strong clustering along the ideal diagonal, confirming minimal prediction error. TCN also reflects a tight distribution. LSTM and GRU scatter plots show wider dispersion.

**Fig. 29:**
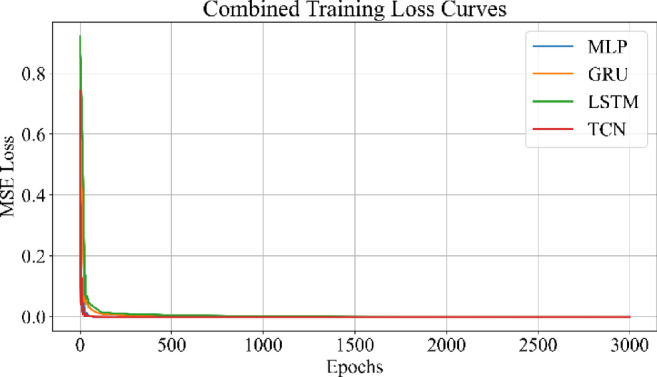
Combined training loss curves for MLP, GRU, LSTM, and TCN models.

**Fig. 30:**
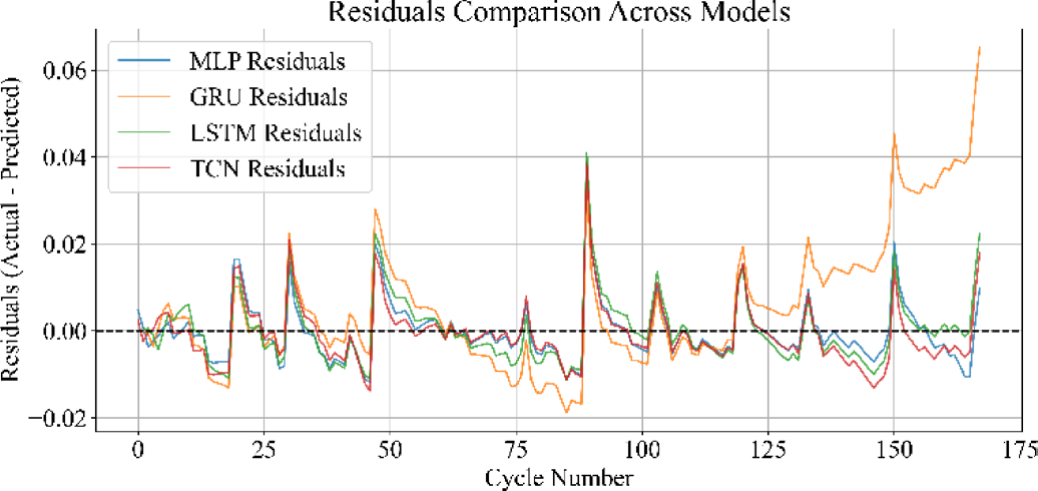
Residuals comparison across all models.

### Training efficiency

Figure 29 shows the training loss curves for MLP, GRU, LSTM, and TCN models. All models reach convergence within 3000 epochs. The MLP demonstrates the fastest and most stable loss reduction, followed closely by TCN, which exhibits similarly smooth convergence behavior. The GRU shows a higher initial loss and slower convergence due to its gating mechanisms and sequential processing overhead. The LSTM follows a similar trend but with slightly reduced computational intensity compared to GRU. These differences in descent characteristics reflect the architectural variations in handling temporal dependencies and parameter update efficiencies.

### Residual distribution analysis

Figure 30 presents the residuals across all cycles for MLP, GRU, LSTM, and TCN models. The MLP shows tightly clustered residuals around zero, indicating minimal deviation from actual SoH values across the dataset. TCN exhibits a similarly narrow spread, with consistent low-magnitude residuals across cycles. LSTM produces slightly more variation than MLP and TCN but remains stable across most of the discharge range. The GRU displays the largest fluctuations, particularly in the later cycles, where residuals become increasingly dispersed. This distribution reflects the relative prediction consistency of each model and highlights the architectural impact on cycle-end accuracy.

### Cross-model comparison of SoH estimation

**Fig. 31:**
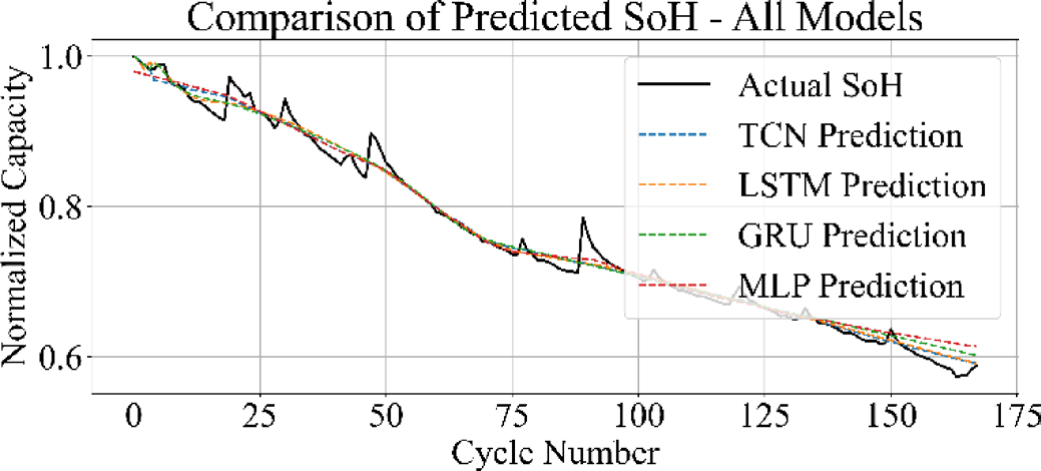
Comparison of predicted vs. actual SoH for all models (GRU, LSTM, MLP, TCN) on the B0006 dataset.

**Fig. 32:**
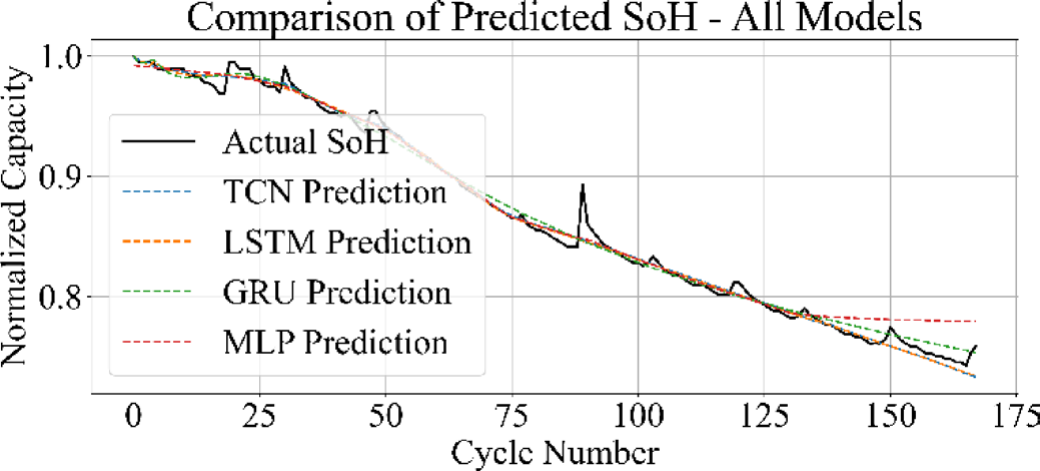
Comparison of predicted vs. actual SoH for all models (GRU, LSTM, MLP, TCN) on the B0007 dataset.

Figures 31 and 32 present the comparative performance of the four models on the B0006 and B0007 datasets. In both cases, MLP and TCN predictions align more closely with the actual SoH trajectory, capturing the overall degradation trend with minimal deviation. The LSTM maintains competitive accuracy but introduces slight underestimation and overestimation near end-of-life cycles. The GRU model demonstrates higher error spread, particularly during the later degradation phase, leading to less consistent predictions.

**Fig. 33:**
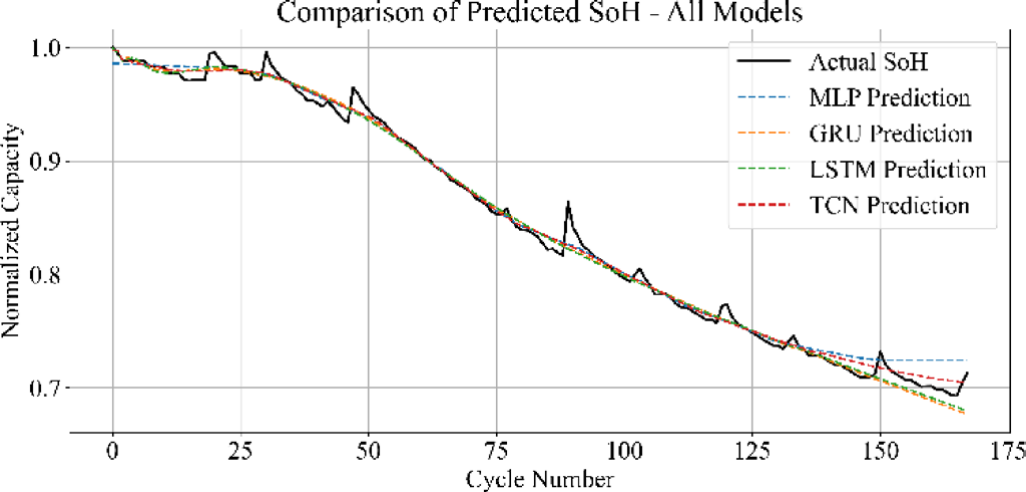
Overall comparison of predicted vs. actual SoH across all models.

Figure 33 displays the SoH estimation trajectories for MLP, GRU, LSTM, and TCN in a consolidated plot. The predicted curves from MLP and TCN align closely with the actual SoH trend, maintaining consistent overlap across all cycles. The LSTM captures the general degradation pattern but introduces slight underestimations in mid-life regions. The GRU predictions exhibit greater divergence, particularly in the final cycles, where the estimated SoH underperforms relative to the true values.

**Fig. 34:**
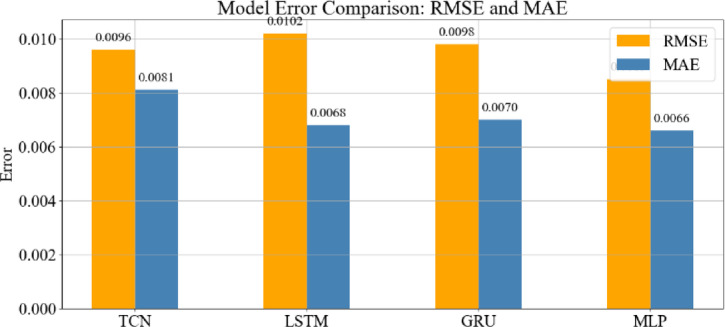
Bar chart of error metrics (RMSE, MAE) for each model.

These results are consistent with the broader benchmarking analysis: MLP achieved the lowest error metrics and fastest training time, followed by TCN, while GRU lagged in both accuracy and efficiency.

### Model error metrics

Figure 34 presents the quantitative error metrics, including RMSE and MAE, for each model. The MLP records the lowest values in both categories, with the TCN performing at a comparable level. The LSTM shows moderate error levels, consistent with its mid-range prediction performance. The GRU exhibits the highest RMSE and MAE, corroborating its visible deviations in the SoH prediction plots and wider residual distribution.

### Key findings

The MLP model demonstrated superior accuracy, efficiency, and generalization, making it suitable for real-time SoH prediction. TCN provided a balance between accuracy and computational efficiency, while LSTM maintained competitive accuracy with moderate computational cost. The GRU, although capable, underperformed in both accuracy and training time. The visualizations presented in this section substantiate the metrics in Table 4 and provide comprehensive insights into model behavior across operational and predictive dimensions.

Cycle-based State of Health (SoH) estimation was conducted using real operational data from the NASA B0005 battery dataset. Four deep learning models such as Multilayer Perceptron (MLP), Gated Recurrent Unit (GRU), Long Short-Term Memory (LSTM), and Temporal Convolutional Network (TCN) were trained and evaluated. Among these, the MLP consistently outperformed other architectures, achieving the lowest RMSE of 0.0069, the lowest MAE of 0.0049, and the highest R² value of 0.9955, all within a training time of just 6.59 s.

The TCN model demonstrated comparable accuracy with an RMSE of 0.0071 and R² of 0.9951, though it required nearly three times more training time than the MLP. Both LSTM and GRU showed acceptable predictive performance; however, the GRU’s training time was significantly higher at 150.06 s, and its accuracy declined relative to the other models.

Loss curves for all models confirmed stable convergence over 3000 epochs, indicating adequate learning across architecture. Residual plots showed tight clustering around zero, suggesting minimal prediction bias and effective generalization across cycles. Scatter plots between actual and predicted SoH further supported these findings, especially for MLP and TCN, where predictions closely followed the ideal line of fit.

From a deployment perspective, the MLP’s rapid convergence and low computational overhead make it highly suitable for real-time integration in embedded Battery Management Systems (BMS). While GRU and LSTM offer competitive learning capability, their recurrent nature results in higher computational demands, limiting their practicality in time-constrained or resource-limited applications. TCN, although slower than MLP, balances accuracy and stability effectively, making it a robust candidate for scenarios prioritizing precision and robustness.

## Conclusion

This research evaluated the performance of four deep learning models as MLP, GRU, LSTM, and TCN—for estimating the State of Health (SoH) in lithium-ion batteries using cycle-based discharge data from the NASA B0005 dataset. The SoH values were computed through numerical integration of discharge current over time and normalized against the initial capacity to capture degradation across lifecycle stages. The models were trained and tested using PyTorch implementations, and their predictive accuracy was assessed using RMSE, MAE, and R² metrics. Among the tested architectures, the Multilayer Perceptron (MLP) demonstrated the highest accuracy, achieving an RMSE of 0.0069, MAE of 0.0049, and R² of 0.9955. The TCN followed closely, with comparable performance (RMSE = 0.0071, R² = 0.9951). Residual analysis confirmed low bias and tightly clustered errors across models, while loss curves exhibited smooth convergence, reinforcing the stability of the training process. The GRU and LSTM models also achieved acceptable accuracy but incurred significantly higher training times due to their recurrent architecture.

The findings indicate that MLP achieved the best trade-off between predictive accuracy and computational efficiency, making it highly suitable for real-time implementation in embedded Battery Management Systems (BMS). The results validate the capability of deep learning models, particularly MLP and TCN, in capturing nonlinear degradation behavior and enabling accurate SoH tracking across the operational life of lithium-ion batteries.

The study evaluated B0005, B0006, and B0007 cells, which share similar chemistries and were tested under controlled laboratory conditions. Results may vary for other chemistries such as NMC or LFP, under different operating temperatures, or under dynamic drive cycles. In this work, models were trained only on cycle-level capacity features; incorporating voltage, current, and temperature time series may further enhance prediction accuracy.

Future research will emphasize the application of transfer learning techniques to extend model generalization across different lithium-ion chemistries, enabling adaptability beyond the datasets evaluated in this study. Incorporation of multi-temperature datasets will be pursued to capture thermal effects on degradation dynamics, thereby enhancing the robustness of SoH estimation frameworks under varied environmental conditions.

## Data Availability

The datasets used and/or analysed during the current study are available from the corresponding author upon reasonable request.

## Abbreviations

SoH: State of health
RUL: Remaining useful life
BMS: Battery management system
GRU: Gated recurrent unit
TCN: Temporal convolutional network
CNN: Convolutional neural network
FPCA: Functional principal component analysis
BERT: Bidirectional encoder representations from transformers
TimeGAN: Time-series generative adversarial network
MAE: Mean absolute error
MSE: Mean squared error
NMC: Nickel manganese cobalt (battery chemistry)
NCA: Nickel cobalt aluminum (battery chemistry)
GPU: Graphics processing unit
cuDNN: CUDA deep neural network library
SoC: State of charge
EV: Electric vehicle
MLP: Multilayer perceptron
LSTM: Long short-term memory
ANN: Artificial neural network
RNN: Recurrent neural network
SETCN: Spectral-enhanced temporal convolutional network
GAN: Generative adversarial network
RMSE: Root mean square error
R²: Coefficient of determination
ReLU: Rectified linear unit
LFP: Lithium iron phosphate (battery chemistry)
CPU: Central processing unit
CUDA: Compute unified device architecture
